# Supplementary material for: Enhancement of Antiviral CD8+ T-Cell Responses and Complete Remission of Metastatic Melanoma in an HIV-1-Infected Subject Treated with Pembrolizumab
Source: J Clin Med. 2019 Dec 1;8(12):2089. doi: 10.3390/jcm8122089 (PMC6947580; doi:10.3390/jcm8122089)
Supplement: Supplementary file 1 [file jcm-08-02089-s001.pdf]

Fig.1 Supplementary

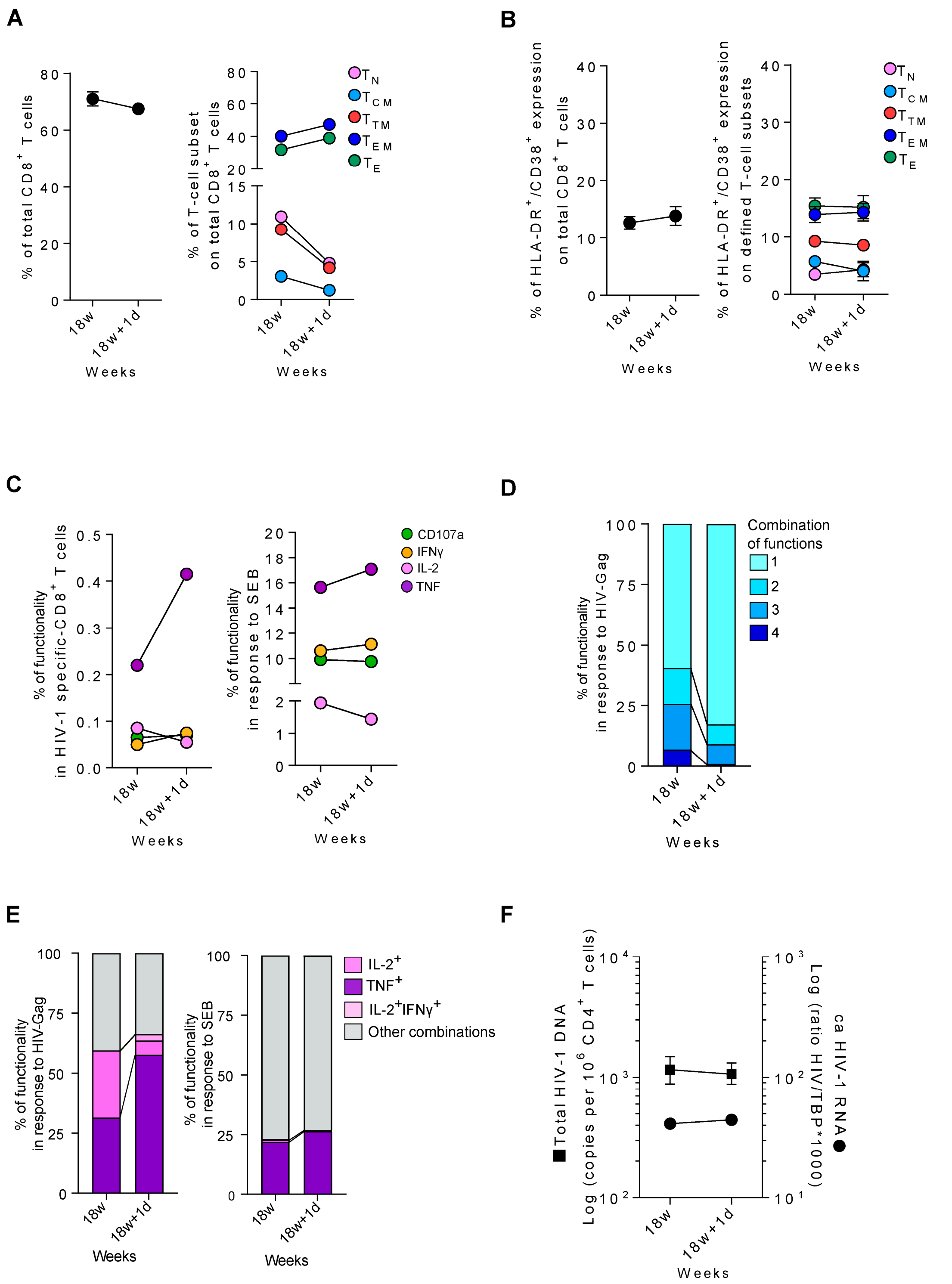

Figure S1. Immunophenotype of the HIV-1 specific-CD8+ T-cell response after 24h of pembrolizumab administration from week 18 (w18+1). (**A**) Changes in total CD8+ T-cell population (left graph) and, in CD8+ T-cell subsets (right graph) after 24h from the administration at week 18. T_N, naïve; T_{CM}, central memory; T_{TM}, transitional memory; T_{EM}, effector memory; T_E, effector; (**B**) The graphs represent the changes in total CD8+ T cells expressing HLA-DR+/CD38+ (left graph) and, in CD8+ T-cell subsets (right graph). T_N, naïve; T_{CM}, central memory; T_{TM}, transitional memory; T_{EM}, effector memory; T_E, effector; (**C**) Changes of TNF,

IFNγ, CD107a and, IL-2 expression for HIV-1 specific-CD8$^+$ T cells in response to HIV-Gag (left graph) and in response to SEB (right graph); (**D**) Frequency of TNFα, IFNγ, CD107a and, IL-2 combinations expressing 1, 2, 3 or 4 cytokines in total HIV-1 specific-CD8$^+$ T cells; (**E**) Detailed poly-functional profiles of HIV-1 specific-CD8$^+$ T cells. The bars in purple represent the changes on TNF frequency and in pink it is represented the changes of IL-2$^+$ and IL-2$^+$IFNγ$^+$. (**F**) Total HIV-1 DNA (squares) and cell-associated HIV-1 RNA (circles) in CD4$^+$ T cells at week 18+1.
